# Development and user-testing of a digital patient decision aid to facilitate shared decision-making for people with stable angina

**DOI:** 10.1186/s12911-022-01882-x

**Published:** 2022-05-27

**Authors:** Emma Harris, Dwayne Conway, Angel Jimenez-Aranda, Jeremy Butts, Philippa Hedley-Takhar, Richard Thomson, Felicity Astin

**Affiliations:** 1 Centre for Applied Research in Health, School of Human and Health Sciences, University of Huddersfield, Queensgate, Huddersfield, HD1 3DH UK; 2 Sheffield Teaching Hospitals NHS Foundation Trust, Sheffield, UK; 3 NIHR Devices for Dignity MedTech Co-Operative, Sheffield, UK; 4 Calderdale and Huddersfield NHS Foundation Trust, Huddersfield, UK; 5 Population Health Sciences Institute, Newcastle University, Newcastle, UK

**Keywords:** Decision aid, Shared decision-making, Patient-centred care, Angina, Coronary angioplasty, Percutaneous coronary intervention

## Abstract

**Background:**

Research shows that people with stable angina need decision support when considering elective treatments. Initial treatment is with medicines but patients may gain further benefit with invasive percutaneous coronary intervention (PCI). Choosing between these treatments can be challenging for patients because both confer similar benefits but have different risks. Patient decision aids (PtDAs) are evidence-based interventions that support shared decision-making (SDM) when making healthcare decisions. This study aimed to develop and user-test a digital patient decision aid (CONNECT) to facilitate SDM for people with stable angina considering invasive treatment with elective PCI.

**Methods:**

A multi-phase study was conducted to develop and test CONNECT (COroNary aNgioplasty dECision Tool) using approaches recommended by the International Patient Decision Aid Standards Collaboration: (i) Steering Group assembled, (ii) review of clinical guidance, (iii) co-design workshops with patients and cardiology health professionals, (iv) first prototype developed and ‘alpha’ tested (semi-structured cognitive interviews and 12-item acceptability questionnaire) with patients, cardiologists and cardiac nurses, recruited from two hospitals in Northern England, and (v) final PtDA refined following iterative user-feedback. Quantitative data were analysed descriptively and qualitative data from the interviews analysed using deductive content analysis.

**Results:**

CONNECT was developed and user-tested with 34 patients and 29 cardiology health professionals. Findings showed that CONNECT was generally acceptable, usable, comprehensible, and desirable. Participants suggested that CONNECT had the potential to improve care quality by personalising consultations and facilitating SDM and informed consent. Patient safety may be improved as CONNECT includes questions about symptom burden which can identify asymptomatic patients unlikely to benefit from PCI, as well as those who may need to be fast tracked because of worsening symptoms.

**Conclusions:**

CONNECT is the first digital PtDA for people with stable angina considering elective PCI, developed in the UK using recommended processes and fulfilling international quality criteria. CONNECT shows promise as an approach to facilitate SDM and should be evaluated in a clinical trial. Further work is required to standardise the provision of probabilistic risk information for people considering elective PCI and to understand how CONNECT can be accessible to underserved communities.

**Supplementary Information:**

The online version contains supplementary material available at 10.1186/s12911-022-01882-x.

## Introduction

Coronary artery disease (CAD) is a common condition affecting upward of 2.3 million people in the UK [1] and over 35 million people in Europe [2]. Angina describes the unpleasant symptoms, such as chest discomfort, that characterise CAD and impact negatively on patients’ health-related quality of life. Initial treatment for angina is with medicines alone, but some patients may gain further symptomatic relief from invasive treatment with elective percutaneous coronary intervention (PCI), also called coronary angioplasty [3]. Both medicines and PCI aim to relieve angina symptoms by widening narrowed coronary arteries through either pharmacological or physical means.

Elective PCI is a low-risk procedure, but heart attack, stroke and death are rare complications [3]. For patients with stable CAD, (with moderate to severe ischemia), elective PCI has been shown to be no better than medicines in reducing the risk of future ischaemic cardiac events or all-cause mortality [3]. Although PCI was more effective in relieving angina than medicines alone for most patients, the clinical outcomes at 5 years were similar [4]. Both treatments aim to relieve angina symptoms but PCI registry data (2018–2019) from the USA showed that 22% of elective procedures were performed inappropriately, with 1 in 6 patients asymptomatic at the time of PCI [5]. Therefore, PCI was unlikely to confer any patient benefit yet exposed them to potential harms. Patients often misunderstand the purpose of elective PCI. They frequently underestimate treatment risks, overestimate benefits, perceive PCI as a ‘fix’ for CAD and are passive in decision-making processes [6, 7].

Involving patients, and those close to them, in health decisions, is an important feature of high-quality person-centred care [8]. Choosing a treatment for stable angina should be a balance between the doctor’s clinical recommendations and the patient’s values and treatment preferences; (i.e. shared decision-making; SDM) [9]. Several countries (Australia, Norway, USA, UK) now include SDM in national health policy and clinical practice guidelines [10–14]. The updated UK General Medical Council (GMC) guidance on decision-making and consent emphasises the importance of involving and supporting patients to make decisions about treatment [10]. There is an increased emphasis on understanding what matters to patients about their health, so that relevant information about the benefits and harms of proposed treatment options can be shared [10]. However, the ‘ideal’ SDM process, outlined in these documents, does not happen consistently in clinical practice and is difficult to implement [15, 16].

Patient decision aids (PtDAs) offer a potential solution as evidence-based interventions that support SDM for healthcare decisions [17]. High-quality evidence from 105 randomised controlled trials show that PtDAs help create better informed patients who are clearer about their health values, have more accurate risk perceptions and are more likely to actively participate in decision-making [17]. International organisations such as the Australian Commission on Safety and Quality in Health Care, UK National Institute for Health and Care Excellence (NICE) and US National Quality Forum recommend the use of high-quality PtDAs in clinical practice [13, 14, 18].

To ensure PtDAs are high-quality, evidence-based, and developed using standardised robust methods, the International Patient Decision Aids Standards (IPDAS) collaboration published a systematic development process and quality criteria [19, 20]. UK SDM guidelines specify that healthcare organisations should use PtDAs that have been quality-assured against either NICE standards or IPDAS criteria [14]. However, there is no high-quality PtDA for elective PCI developed using this approach that we are aware of for UK use. Existing PtDAs for elective PCI are either outdated [21], unavailable (commercial licence required or no longer accessible) [22–25], developed and tested outside the UK [21–23, 25] and/or do not meet the required quality assessed IPDAS criteria [21–25]. In controlled trials, two existing PtDAs were shown to improve patients’ knowledge of treatments and outcomes, but did not improve SDM and were difficult to implement in clinical practice [22, 26]. The long-term implementation of PtDAs is challenging, but evidence collected over an 8-year period suggests that integrating them into electronic patient records helps to increase adoption [27]. This study describes the development of a digital PtDA called **CONNECT (CO**ro**N**ary a**N**gioplasty d**EC**ision **T**ool) and presents results from user-testing.

## Methods

### Overview of study design

**Fig. 1:**
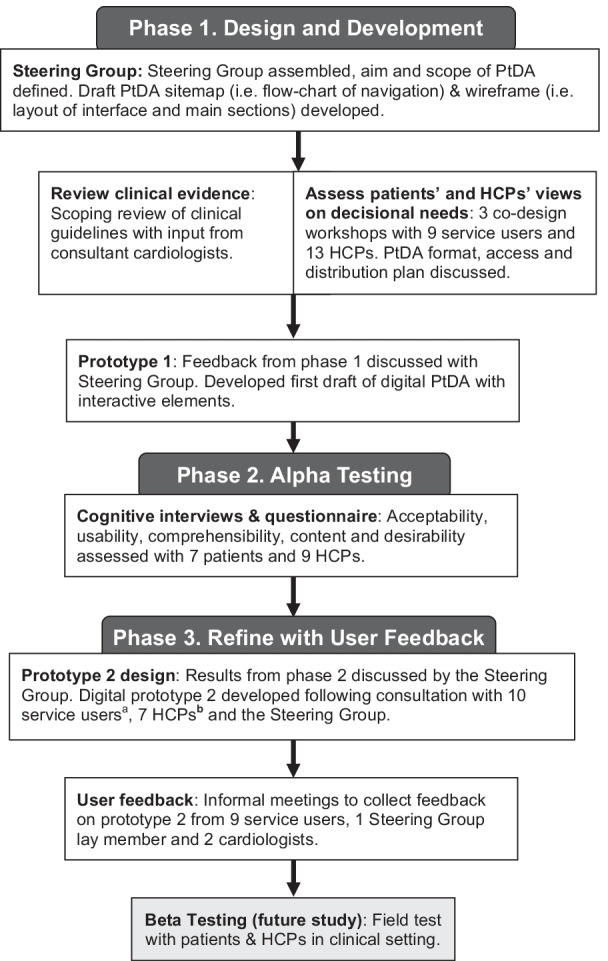
Systematic development process of CONNECT, based on the IPDAS PtDA development model [19]. HCP: healthcare professional; PtDA: patient decision aid; service users refers to volunteers from UK heart support groups. ^a^Three volunteers had taken part in the first co-design workshop. ^b^Three cardiologists and 4 cardiology specialist nurses; 5 were involved in the previous phases

The study comprised three phases (outlined in Fig. 1), which were informed by the IPDAS systematic development process and quality criteria [14, 19, 20]. The Ottawa Decision Support Framework provided a theoretical foundation for the process; it states that PtDAs improve decisional quality by addressing a patient’s unresolved decisional needs [28]. Treatment decisional needs is a deficit in what a patient requires when making a high-quality decision; i.e. one which is informed and aligns with patients’ health values and treatment preferences [28]. To ensure that CONNECT addressed previously identified unmet decisional needs [6, 7], phases one and three were underpinned by the principles of co-design; a user-centred design method that involves researchers, designers and end-users (patients, health professionals) actively working together in partnership to create a product or service [29]. Phase two (alpha testing) adopted a descriptive qualitative research design, involving semi-structured cognitive interviews and questionnaires. Alpha testing refers to preliminary user-testing of PtDAs to evaluate acceptability, usability and comprehensibility [19]. The Standards for UNiversal reporting of patient Decision Aid Evaluation studies (SUNDAE) guided the reporting of this study (Additional file 1: Table S1) [30].

### Setting and context

The study was conducted in Northern England and health professionals were recruited from two District General Hospitals. The ‘typical’ patient pathway for people with stable angina involves referral for diagnostic coronary angiography with the option to proceed immediately to PCI treatment, during the same procedure, if clinically indicated. Ahead of hospital admission, patients have a face-to-face, telephone or video consultation led by a specialist cardiac nurse.

### Phase 1: Design and development of CONNECT

#### Steering Group and PtDA scope

A multidisciplinary Steering Group was convened at the study outset to work with the research team to confirm the aim and scope of CONNECT and guide its development (2 lay members, 2 consultant interventional cardiologists, 1 cardiac specialist nurse, and 1 expert in SDM and PtDA development). The aim of the PtDA was to provide decisional support for patients experiencing symptoms of stable angina and deciding whether to continue with cardiac medicines alone, or to progress to invasive treatment with elective PCI plus medicines. To link with electronic patient records it was agreed that the first version of CONNECT would be a web-based and mobile application (App) accessible on all digital devices.

#### Review clinical evidence

A search of UK NICE, British Cardiovascular Intervention Society (BCIS) and European Society of Cardiology (ESC) clinical guidelines and websites identified content for the sections that described treatment options and associated risks and benefits. Two practicing consultant cardiologists reviewed clinical evidence, which included NICE management guidelines for stable angina [31], 2019 ESC guidelines for chronic coronary syndromes [32], and BCIS 2017–2018 PCI audit data [33].

#### Assessment of patients’ and healthcare professionals’ views on decisional needs

Three face-to-face participatory co-design workshops were convened to identify patients’ decisional needs and how CONNECT could be best designed to address them. One 3-h workshop, (facilitated by two researchers at the University of Huddersfield), was attended by nine ‘expert patients by experience’ who had undergone coronary angiography or PCI. Volunteers were recruited through online adverts posted across the University and community ‘Heart Support’ groups. Two 90-min workshops were run at two NHS Trusts in Northern England and attended by thirteen health professionals (4 cardiologists and 9 nurses). Field notes were recorded by two researchers to summarise patients’ and health professionals’ decision support needs, views about CONNECT’s draft sitemap and wireframe, format, access and distribution plan, graphics, preferences for risk communication and preferred methods for eliciting patient’s health values and goals. Potential factors that could influence CONNECT implementation were also discussed such as differences in the patient pathways between hospitals. Workshop activities and how the feedback informed the CONNECT prototype 1 are detailed in Additional file 1: Table S2.

#### Prototype 1

**Fig. 2:**
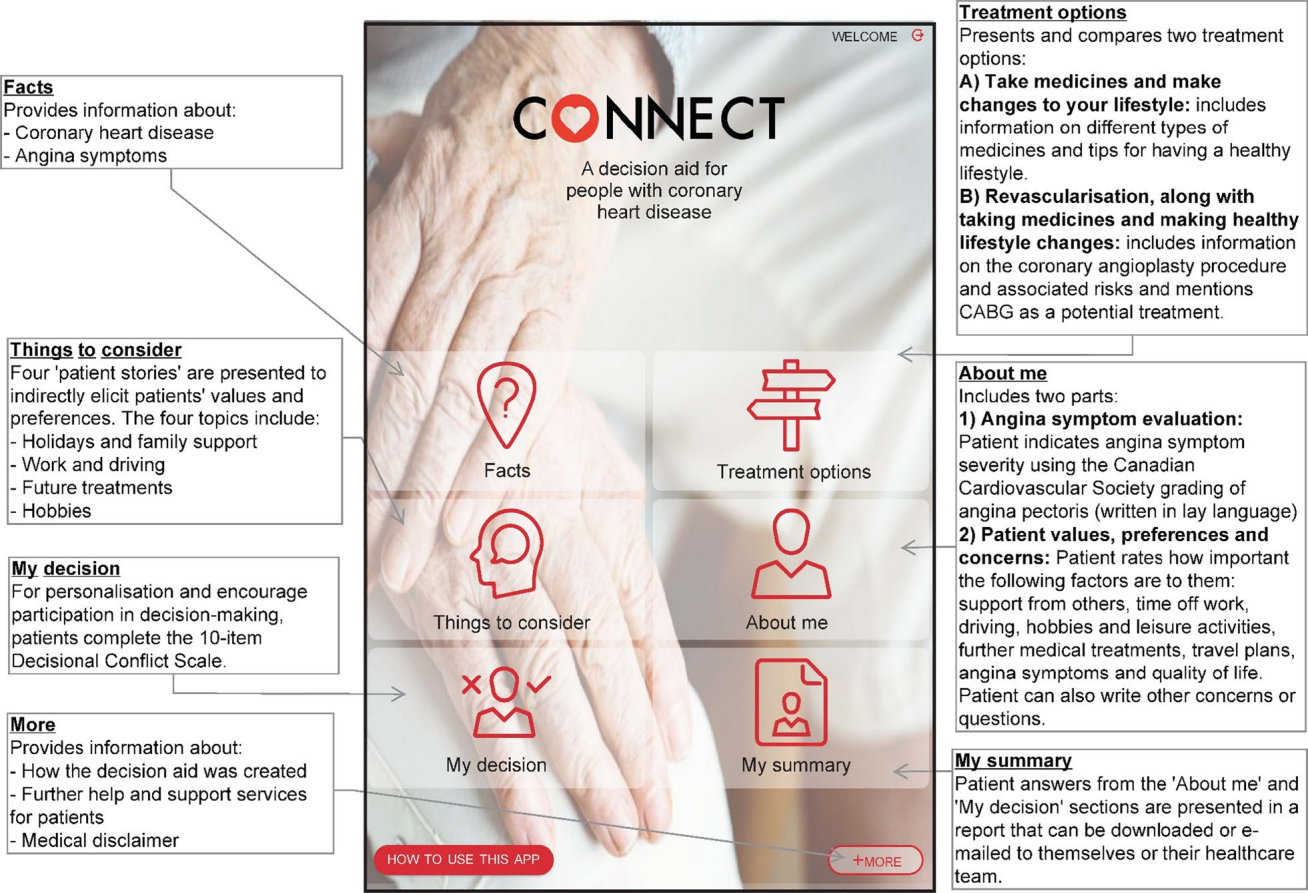
CONNECT prototype 1: ‘home’ screen and outline of main sections

The first digital prototype of CONNECT (Fig. 2) was produced following an iterative process driven by Steering Group discussions, feedback collected from the co-design workshops and the IPDAS standards [20]. The intention is for CONNECT to be made available to patients at the point of referral for PCI via a digital link and/or QR code in a text, e-mail, letter or flyer. CONNECT was designed to be accessed by patients at home before the pre-assessment consultation with a specialist cardiac nurse that occurs before hospital admission. One innovative aspect of CONNECT was the built-in functionality that generated a personal summary of patients self-reported angina symptoms, values, treatment preferences, worries, concerns and questions to ask the nurse at the pre-assessment consultation. This summary was designed as a ‘primer’ that could encourage personalised discussions between the patients and specialist cardiac nurse to facilitate SDM.

### Phase 2: Alpha testing

The aim of alpha testing was to evaluate the acceptability, usability, and comprehensibility of CONNECT prototype 1 in non-clinical settings with patients and health professionals. Perspectives on CONNECT’s content and desirability were also explored.

#### Participants

Between 10 and 20 participants are required to find 95% of all website usability problems [34]. We aimed to recruit simultaneously a purposive sample of up to 20 participants (10 patients and 10 HCPs) from two hospitals in Northern England.

All patients receiving PCI treatment, within the previous 18 months, who were able to speak and read English, and had no cognitive impairment, were eligible to participate. Attendant cardiologists identified eligible participants and provided them with study information.

All cardiologists and specialist nurses who had experience of providing healthcare to patients preparing for elective PCI were eligible to participate. Those previously involved in CONNECT development were excluded. A Research Nurse provided eligible participants with study information and invited them to contact the research team if they wished to participate. Informed consent and data collection took place at either the participant’s home or in a private room at the University of Huddersfield or hospital.

#### Cognitive interviews and questionnaires

CONNECT prototype 1 was evaluated through individual face-to-face cognitive interviews and a researcher-generated 12-item acceptability paper questionnaire. Cognitive interviewing is an evidence-based qualitative method that focuses upon how individuals mentally process and respond to a task [35], and is commonly used to ‘alpha test’ PtDAs [36–39].

During the cognitive interviews, participants were observed logging into, and using, CONNECT prototype 1 on an iPad by a researcher (EH). Field notes were recorded about the ease of iPad handling, App navigation and completion of interactive tasks, as well as non-verbal communication and any assistance patients required from friends/family. Cognitive interviews adopted the ‘verbal-probing’ approach [35]; participants were asked proactive (pre-determined) questions after each section of CONNECT was read/completed. Questions were adapted from other published studies [36–39] to explore participants’ perspectives on the acceptability, usability, comprehensibility, desirability and content of CONNECT (see Additional file 1 for the interview guide). If any issues emerged during the use of CONNECT, questions (reactive probes) were asked. Interviews were audio-recorded and transcribed verbatim.

Following the interview, participants completed the 12-item researcher-generated acceptability questionnaire (see Additional file 1). The measure was adapted from the validated ‘Acceptability E-scale’ [36], which included Likert scale responses, to evaluate acceptability, usefulness, understanding and satisfaction. Patient participants’ health literacy level was measured using the validated three-item Brief Health Literacy Screening Tool [40].

#### Data analysis

Quantitative data from questionnaires were analysed descriptively using SPSS software (IBM SPSS Statistics 24). Qualitative data from the interview transcripts were managed and retrieved using NVivo software (Version 12; QRS International Pty Ltd) and analysed using deductive content analysis, following a 5-stage unconstrained matrix approach (see Additional file 1: Table S3 for further details) [41]. Trustworthiness of the qualitative analysis was supported by investigator triangulation (two researchers developed the categorisation matrix) and the recording of detailed observations during interviews. Emerging findings were discussed with the research team during the analytical process to support an on-going process of reflexivity. A detailed account of the study methods supports the confirmability of the findings.

### Phase 3: Refine with user feedback

#### Participants

Volunteers for the prototype 2 design and user-feedback consultations were recruited through snowball (referral from participants and Steering Group members in previous phases) and convenience sampling (members of UK heart support groups responding to e-mail invitations/adverts). Support group members were eligible to volunteer if they were ‘experts by experience’ through living with or caring for a person with CAD. Cardiologists and specialist nurses whose current or previous role involved contact with patients prior to elective PCI, were eligible to participate.

#### Prototype 2 design

The CONNECT prototype 1 was refined using findings from alpha testing in an iterative process involving remote video consultations (up to 1-h duration) with the Steering Group, 10 members of community heart support groups and 7 cardiology health professionals (see Fig. 1). The consultation process led to the inclusion of a revised angina symptom evaluation questionnaire, an extra patient story, an updated value clarification method (animated video plus ‘field’ for patient to record their values), a simplified decision preference section and new multimedia. Data from a systematic review published during the development of CONNECT on patient outcomes in people with stable angina following medicines, or PCI, updated the section providing average population risk estimates [42]. Updated functionality enabled the personal summary section to be saved, printed or shared via e-mail, to help with future integration into electronic patient records.

**Fig. 3:**
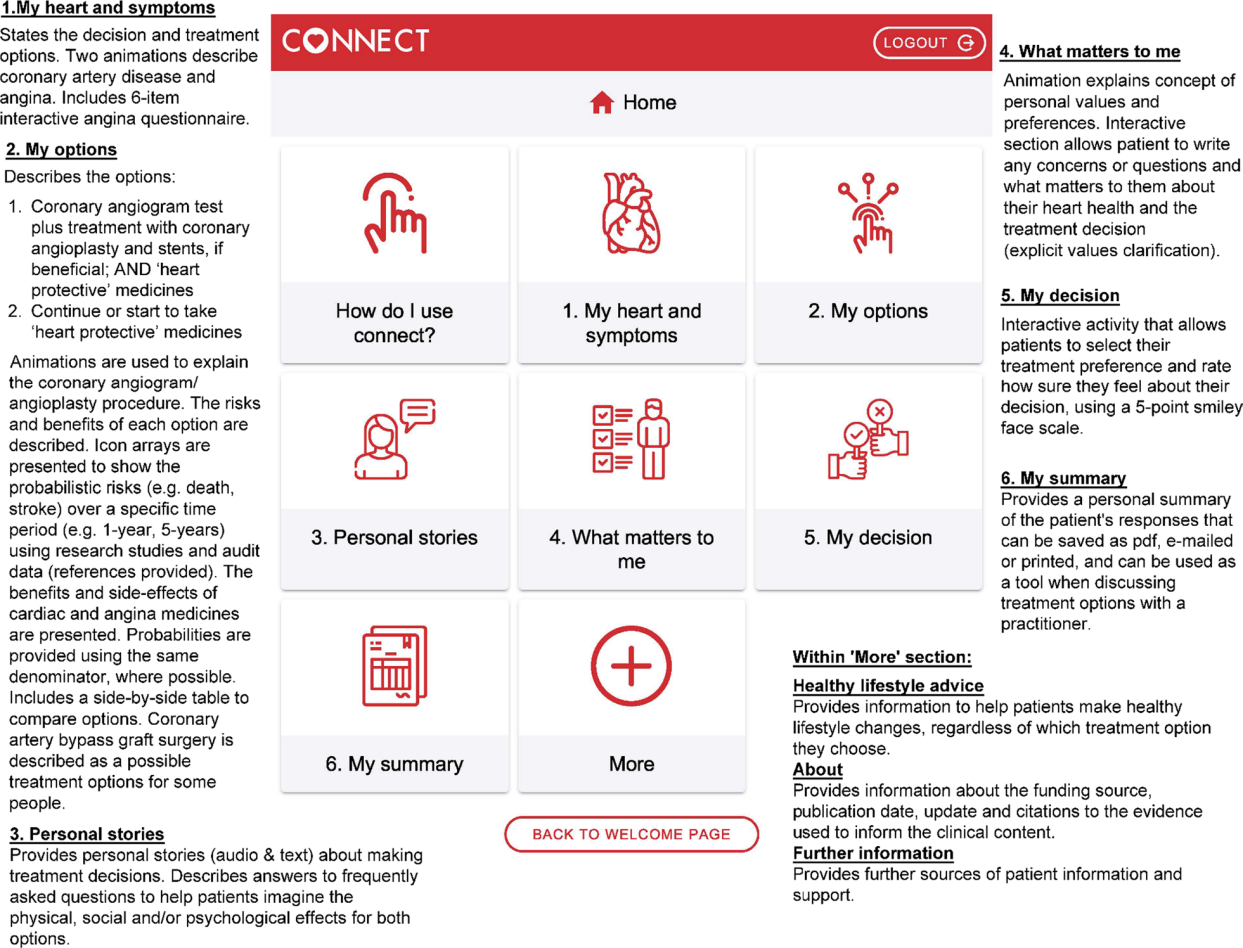
CONNECT prototype 2: 'home' screen and overview of main sections

Following several iterations, CONNECT prototype 2 was finalised and achieved all of the 12 applicable mandatory qualifying and certification criteria of the IPDAS version 4 PtDA checklist [20]. Sixteen of the 23 optional IPDAS quality criteria were also achieved (Additional file 1: Table S4). Field testing and effectiveness criteria were not applicable as they will be evaluated in the future beta testing phase. Figure 3 provides an overview of CONNECT prototype 2. See Additional file 1 for screenshots of CONNECT prototype 2 (the full version is available for free upon request from the authors).

#### User feedback

To collect feedback on CONNECT prototype 2, further remote video consultations (up to 1-h duration) were conducted by one researcher (EH) with 9 new volunteers from community heart support groups, 1 Steering Group lay member, and 2 consultant cardiologists not involved in previous study phases. The website link to CONNECT was sent to volunteers who were asked to consider the presentation, comprehensibility, navigation, and acceptability of CONNECT. Key points raised in the consultations were recorded in field notes, discussed with the research team and summarised as a narrative.

## Results

### CONNECT prototype 1 alpha testing

Seventeen participants (6 patients, 1 patient/partner dyad and 9 health professionals) completed alpha testing. Participants’ demographics are detailed in Table 1. All items of the acceptability questionnaire were rated highly (Table 2). The mean (standard deviation) duration of interviews was 77 (17) minutes. Content analysis of the cognitive interviews identified 5 themes and 14 categories (Table 3). Representative participant quotes for each category are shown in Table 3 and further quotes for each code are show in Additional file 1: Table S5.

**Table 1:** Alpha testing participant demographics

	Patients (n = 7)	HCPs (n = 9)
Age (years)		
Mean (SD)	63 (11)	42 (6)
Range	50–76	26–58
Female [% (n)]	29 (2)	44 (4)
Role	–	4 Cardiologists
		4 Cardiology specialist nurses
		1 Interventional radiology matron
Years practicing speciality [mean (SD)] n = 8	–	12 (8)
Level of education [% (n)]		
High school [up to 16 years old]	14 (1)	–
College [16–18 years old]	14 (1)	–
University or equivalent [18 + years]	71 (5)	–
Type of angioplasty [% (n)]		
Elective	71 (5)	–
Urgent	29 (2)	–
Health literacy (HL) level* [% (n) participants]		
Marginal HL (score between 5 and 8 out of 12)	29 (2)	
Adequate HL (score between 9 and 12 out of 12)	71 (5)	

^*^Scores of between 0 and 4 indicates limited/lowest HL, 5 and 8 indicates marginal HL, and 9 and 12 indicates adequate/highest HL levels

**Table 2:** Alpha testing acceptability questionnaire results (n = 16)

Questionnaire item	Median (interquartile range)^a^
1. How easy was it to use CONNECT?	4 (4–5)
2. How understandable was the information?	4 (4–5)
3. How much did you enjoy using CONNECT?	4 (4–5)
4. How helpful was CONNECT?	5 (4–5)
5. Was the amount of time it took to complete CONNECT acceptable?	4.5 (4–5)
6. Overall, how would you rate your satisfaction with CONNECT?	5 (4–5)
7. Please rate how useful you found the ‘Facts’ section	5 (4–5)
8. Please rate how useful you found the ‘Treatment options’ section	5 (4–5)
9. Please rate how useful you found the ‘Things to consider’ section	4 (4–4)
1.0 Please rate how useful you found the ‘About me’ section	4 (3–4)
11. Please rate how useful you found the ‘My decision’ section	4 (3.75–4.25)
12. Please rate how useful you found the ‘My summary’ section	4 (4–5)

^a^Each item of the questionnaire was scored on a 5-point Likert scale, where 1 was the lowest possible rating and 5 was the highest

**Table 3:** Alpha testing illustrative participant quotes

Themes	Categories	Illustrative quotes
1. Acceptability	1.a. Facilitating shared decision-making	**Nurse 12:** “It’s just empowering them isn’t it and it’s giving them, well it’s giving them a voice, it’s allowing them to actually make a choice without just hearing one side of it from a doctor. It’s actually allowing them to think about, well first of all its actually letting them know that they have a choice, it isn’t just you need a stent and then it’s letting them be able to have the knowledge about both options and maybe with the help of doctors and nurses, come to a decision”
	1.b. Improving care processes	**Nurse 10:** “If they’ve got these things to discuss, it would make the pre-assessment a bit more like personal to each patient, if you had this before pre-assessment, you’d be like right I’m going to talk to them about this, this and this. Whereas at the moment you kind of have a bit of a spiel and then you go off what the person kind of says back to you and if they’ve been able to do this at the, in their home, rather than doing a questionnaire or anything when you’re there with them, they’re being a bit more honest maybe and I like the score”
	1.c. Quality and safety in practice	**Cardiologist 1**: “I think the benefit of this, if you get the patient to fill it in, it might actually start to make some doctors think are we actually doing the right thing for this patient? So, I think for the wrong reasons it might have the right effect”
2. Usability	2.a. Accessibility	**Patient 5:** “The only slight downside and I’m thinking of my mum here, she can be very IT averse and can feel threatened by it. But to me it’s, its straightforward and easy enough. Whereas someone a bit older might, well not older, but IT averse”
	2.b. Navigation	**Patient 6:** “Maybe some, yeah, quick navigation buttons to get you back to the home page menu, main menu, something like that”
	2.c. Functionality	**Cardiologist 4:** “It would be helpful, yeah, that would be helpful. Yeah, so I think what you’re saying is that yeah, exporting this output is really helpful. Whether email is like an option is open to debate. So, I know, you know, if there’s an option to export to pdf and then I don’t know, somehow send it in such a way that it’s just attached to the electronic patient record or something, then that’s really useful”
3. Comprehensibility	3.a. Language	**Patient 6:** “Again it’s explaining all in nice straightforward non-technical terms, but then starts then giving me some names that I can’t say, atherosclerosis”
	3.b. Interpretation of information	**Researcher:** “What do you think of having the picture there with the people?” **Patient 7:** “Oh it’s easier than saying one in three thousand or something like that, you might as well just look at it and say I might be him” **Patient’s partner 7:** “If you’re not mathematically inclined that’s quite a good way of doing it, you know, you think what’s one in one hundred, no that’s rather good that”
4. Content	4.a. Accuracy of content	**Cardiologist 1:** “That’s just wrong. One in a thousand. The BCIS wouldn’t quote that. It’s about one in a hundred. So that’s, that to me looks as if there’s no risk, doesn’t it?”
	4.b. Balanced view of treatment options	**Nurse 3:** “I like that side by side, I thought that was quite good how you could do the little snapshot. I know it wasn’t in as much detail as the bigger options, but you could see side by side as like a, you’re doing it to make a choice aren’t you, so it’s showing you the pros and cons of each one and how they might fit for you”
	4.c. Personalisation of decision aid	**Patient 14:** “Well it’s good to have it all as a summary, having been through the bits and perhaps forgotten about some of them and having the list of questions written down”
	4.d. Value elicitation method	**Patient 9:** “I think that might be quite good, that use this space to write down any concerns or questions that you want to discuss with your doctor. That might be, that might focus your mind to actually prepare yourself for a visit to the doctor so that you actually have your questions. I mean sometimes it’s a good idea just to write them out yourself”
5. Desirability	5.a. Presentation	**Cardiologist 15:** “Maybe the text should be bigger because its, I don’t know, for elderly patients, they might struggle to read some of the smaller, I guess it depends how big the screen is doesn’t it? Maybe it’s a bit small, the text, for older patients to read. But nice setup”
	5.b. Use of multimedia	**Patient 2:** “The visual aspect there just leaps out straight away and says right that’s the problem you’ve got, there it is, that’s what we do and that’s, once the balloon is taken out, it's left in as that”

#### Acceptability

This theme refers to the ways in which CONNECT could potentially add value to patients, health professionals and health services. Both patient and health professional participants identified three important potential benefits of CONNECT. Firstly, participants felt that CONNECT had the potential to improve SDM through the provision of consistent, trustworthy information ahead of pre-assessment clinic consultations. Knowing that CONNECT provided trustworthy health-information was reassuring and could potentially save patients’ time spent using ‘Google’ to access health-information. All participants felt that using CONNECT would encourage greater involvement, make people aware that they had a choice, better inform patients, and potentially increase confidence about their treatment decision. Paradoxically, most patient participants still felt that the treatment decision was the doctors to make, as the experts. Some health professionals agreed that patients don’t want to make the choice themselves. Being able to work through CONNECT at their own pace and revisit health information at leisure was perceived as advantageous and a way of improving recall. Content that helped patients to understand how the different treatment options might affect their lives and elucidate personal preferences and concerns was particularly valued, because such topics were often missed in consultations due to time constraints. Concerns were raised that CONNECT may not be acceptable to people with lower levels of education.

Secondly, CONNECT had the potential to improve care processes such as the personalisation of pre-assessment clinic consultations and informed consent. Participants felt that CONNECT could raise health professionals’ awareness and recognition of common patient misunderstandings about PCI treatment and equip patients with important health-information ahead of consultations. Participants particularly valued the personal summary function, as a way of potentially saving consultation time by enabling the cardiologist/nurse to tailor the discussion to be more personalised to the patient’s needs.

Thirdly, several health professionals suggested that the angina symptom questionnaire had the potential to enhance patient safety. This could occur through avoiding unnecessary PCI procedures for patients who no longer have angina symptoms, or by identifying patients who experience a sudden increase in angina severity, and need to be fast-tracked for urgent treatment. A few participants identified the potential benefit of linking CONNECT with electronic patient records to both evidence and audit the quality of the informed consent process.

#### Usability

This theme refers to how well CONNECT was accessed, navigated, and completed by participants. Several issues were raised about the accessibility of CONNECT. The login process was a challenge for half of the participants due to the format of the ‘date-of-birth’ field. Despite this, all participants were able to use CONNECT unaided. However, most participants felt that patients with low levels of digital literacy would have difficulty accessing and using a digital PtDA, without support from family, friends, or health professionals. It was suggested that older patients, people with lower education levels, and those with English as a second language would be particularly disadvantaged. Conversely, the patient participant with the lowest self-reported education and health literacy level, felt that people with low digital literacy skills would find CONNECT easy to use. Despite these concerns, the interactivity of CONNECT was generally viewed as superior compared to paper-based resources.

There were several aspects of the navigation, which needed improvement and suggestions were made about additional functions to increase user interactivity and personalisation. The ability to export the ‘My summary’ was positively received. However, concerns were raised about the transfer of confidential information and potential burden from high volume of e-mails.

#### Comprehensibility

This theme describes participants’ understanding of CONNECT. Most participants felt that the language used within CONNECT was concise and easy to understand, although both patients and health professionals identified some medical terminology that required simplification. Two health professionals questioned whether people with lower education levels or English as a second language would find the information accessible.

The visual presentation of risk using icon arrays was unanimously supported as an approach to improve patient understanding of the probabilistic risk of PCI complications. A minority suggested that icon arrays for the less common risks could be misleading.

Two aspects of CONNECT were identified as difficult for participants to understand and interpret; the decision scores (calculated from patient answers to the Decisional Conflict Scale) and the instructions for interactive features. Participants suggested the addition of instructions about the purpose of each section and how to complete the interactive elements.

#### Content

This theme provides participants’ perspectives on the usefulness and factual accuracy of CONNECT’s content. Participants suggested improvements to the content within all sections of CONNECT. The information on medicines and lifestyle changes was positively reviewed. Minor changes to dietary information were recommended to reflect different cultures and ethnicities. There was no consensus amongst cardiologists about the precise probabilistic risk information that should be included. The inclusion of trial evidence suggesting that planned PCI would not reduce the risk of future cardiac events was not fully supported by all cardiologists.

The side-by-side presentation of treatment options helped to provide a balanced view, but a mismatch in the proportion of content of PCI versus medicines alone could introduce bias. Some participants from both patient and health professional groups mentioned that coronary artery bypass surgery should be included as a treatment option.

The personalised features of CONNECT were generally well received. Most participants supported the inclusion of an angina symptom questionnaire and personalised summary section. Changes to the description of angina symptoms and format of the questionnaire were suggested by both patients and health professionals.

The validated decisional conflict scale received a mixed response across participants groups. Most felt that it would be useful, but others perceived it to be a check-list exercise with one patient disliking the idea of being ‘tested’. The features used to promote reflection and discussion about patients’ values and preferences were supported by many participants. Most patient participants felt able to relate to the ‘Patient Stories’ but suggested to include a younger patient in the next prototype. The opportunity to complete value statements and the ‘concerns’ field was generally positively received. Health professionals thought this would be useful to highlight patients' perspectives.

#### Desirability

This theme refers to the degree to which CONNECT was presented in a visually appealing way. Overall, CONNECT was visually appealing to participants with only minor changes suggested. Most participants suggested a larger text size for those with visual impairments. The simple colour scheme was generally well received as were the style of the icons/buttons and layout. The images and videos were positively reviewed, and the inclusion of additional multi-media was suggested to reduce volume of text.

### CONNECT prototype 2 user feedback

Positive feedback was received from all volunteers. They felt CONNECT prototype 2 was clear, well presented and easy to access. Although some improvements were suggested (e.g. larger text size) before ‘beta testing’, the animations and illustrations were simple and informative and the language used in CONNECT was understandable to all volunteers.

Preferences for risk presentation varied; some preferred numerical frequencies and others preferred the visual icons. Most felt that the icon arrays would support patient understanding of risk estimates. Several heart support group members and one cardiologist felt that risks should be presented in order of severity; others felt that the current presentation (most common to least common) was appropriate.

Most volunteers found CONNECT easy to navigate and believed it would help patients feel more involved in the decision-making process. All agreed that if they were given CONNECT as a patient considering elective PCI, they would complete the interactive sections and would use the personal summary to help prepare for a consultation.

## Discussion

This is the first study to provide a detailed description of the multi-phase development process and user-testing of a digital PtDA for stable angina patients, developed in the UK using recommended processes and fulfilling international quality criteria. The use of comprehensive PtDA user-testing methods ensured that CONNECT reflected the needs of patients and health professionals, and identified where it could be feasibly integrated into the planned PCI patient pathway. Findings showed that CONNECT was generally acceptable, comprehensible, usable, and desirable with only minor changes to the content required before evaluation (‘beta testing’) in clinical settings. A strength of the study is that CONNECT achieved all of the mandatory IPDAS quality criteria for PtDA development and content [14].

Novel features of CONNECT included personalised self-report sections that enabled patients to indicate their angina symptom burden, specify their preferred treatment option, list factors considered most important to them when making their decision and note any concerns or questions. These data generate a personalised summary to be used to inform future consultations with health professionals. The summary could be integrated into the electronic patient records, an approach known to support the implementation of PtDAs into health services [27]. The features of CONNECT were perceived to offer several potential benefits.

CONNECT has the potential to help facilitate SDM and improve informed consent, by providing health information relevant to the decision in advance of a consultation using an engaging and interactive format. Patients are more likely to understand medical procedures and associated risks and benefits when information is presented in an interactive digital format, compared to written or audio-visual [43, 44]. Better informed patients may have fewer misconceptions about treatment outcomes. This is particularly important in this patient group who often mistakenly perceive PCI as curative [6, 7]. Moreover, international guidelines advocate for improvements in SDM and informed consent processes, but provide much less detail about how this might be achieved in clinical practice [10–14]. The PtDA CONNECT offers potential solutions. Health information and preference elicitation activities enable the patients to deliberate about possible treatment options ahead of consultations. The summary of patient’s health values, concerns and treatment preferences, shared with health professionals ahead of a consultation, may act as a ‘primer’ to promote more personalised discussions about what matters most to the patient.

CONNECT may improve patient safety. Planned PCI has been performed unnecessarily at times, exposing patients to avoidable harms [5]. The presence of angina symptoms is the main driver for elective PCI treatment. Therefore, the inclusion of the angina symptom questionnaire that can be reviewed at pre-assessment clinics, may help to identify asymptomatic patients who are less likely to benefit from PCI treatment. Conversely, ongoing assessment of worsening angina symptom burden may fast-track PCI for those most in need.

The development of CONNECT highlighted important learning for the future implementation and development of PtDAs across international health settings. User-centred design is an essential element of the IPDAS systematic PtDA development model, yet there is no consensus around the best methods to use and considerable variation in the level of user involvement [19, 45]. Gathering perspectives on early drafts (storyboards, wireframes), conducting preliminary evaluations via interviews, focus groups and surveys, using cognitive interviews to observe PtDA interactions and including lay members in advisory groups are suggested approaches [45]. We adopted all of these approaches and found remote video consultations to be a particularly useful tool to gather feedback from individuals across the UK. To support UK and international PtDA developers to incorporate user-centred design methods, we have included the cognitive interview guide, acceptability questionnaires and a detailed description of the co-design workshop activities in the additional file.

An important finding from alpha testing was the tension between the patient’s desire to be more involved in decision-making after using CONNECT, coupled with hesitation about voicing their preferred treatment option. Previous studies report that patients understandably view cardiologists as the experts and trusted their treatment recommendation [7]. To shift from this ‘paternalistic’ to a ‘shared’ perspective of treatment decision-making, requires more than providing a PtDA alone. Research shows that patients need health professionals to explicitly ask them to use a PtDA and share their values and preferences, before feeling like they have permission to become involved in decision-making [46]. Moreover, health professionals often perceive that SDM is already provided in consultations, even though observational data show that this is not always the case [47]. At an organisational level the appropriate infrastructure is needed to support and embed SDM in patient pathways. Training and support in SDM skills is needed for the health professional workforce, to enable them to strengthen SDM approaches in their clinical practice [46]. To promote patient engagement, IPDAS recommends that PtDAs and accompanying information are sent to patients ahead of consultations [46]. During the subsequent consultation, health professionals could remind patients to use the PtDA, to help encourage them to share their values and treatment preferences [46]. SDM skills workshops and PtDA introductory training sessions with the whole team are recommended to help health professionals understand differences between current practice and SDM and identify how the PtDA can be integrated into existing care pathways [46]. The implementation plan for CONNECT aligns with these recommendations. Training sessions about CONNECT and SDM will be provided to cardiology teams. Patients will receive invites from their healthcare team to access and use CONNECT. Accompanying information will focus on encouraging patients to complete and share their personal summary with the nurse facilitating their PCI pre-assessment clinic appointment. We aim to test the feasibility of implementing CONNECT using this approach in a future study.

Best practice guidelines for PtDA development and IPDAS quality criteria recommend the inclusion of probabilistic estimates for risks of possible treatment complications [20, 48]. Although there is little consensus on the optimum presentation, some evidence shows that visual risk formats (i.e. graphs, icon arrays) lead to more accurate risk perception [49]. Patient/service user and health professional preferences for risk presentation varied in our study; some preferred the visual icon arrays whereas others preferred the numeric frequencies. Providing multiple formats is perhaps the best option to account for differences in individuals’ numerical literacy levels and preferences [50]. Additionally, the use of ‘teach-back’ to check how well a patient understands health information may be a useful skill for health professionals to master [51]. Teach-back involves asking patients to explain information ‘back’ to the health professional in their own words, which is a useful way to evaluate the effectiveness of a patient’s education session. Teach-back can improve patients’ understanding of procedural risks, benefits and alternative treatments [43]. Presenting standardised risks and benefits in PtDAs in combination with ‘teach-back’, may be a promising way forward to improving risk communication.

The lack of standardised population risk estimates for PCI is a global challenge for health professionals, patients and those close to them. There was no consensus amongst cardiologists regarding the average population estimates for PCI procedural risks communicated to patients. The lack of consensus on the ‘correct’ numerical risk to communicate is observed across other medical specialities in several countries [52]. Health professionals often underestimate risks and overestimate benefits indicating a need for support with this challenging aspect of a consultation [52]. Alternatively, patients and policy makers may need to accept that health professionals cannot always be certain about the risks and benefits of a procedure. Patient responses to expressions of uncertainty are variable, having either positive or negative effects on satisfaction, anxiety, SDM and trust in practitioners [53]. Health professionals should carefully consider how they communicate uncertainty about health outcomes. Disclosure of uncertainty is more likely to have a positive effect when the practitioner is confident, calm, reassuring, open and empathic [53, 54].

Health and digital literacy levels are also important considerations when developing risk and other health-related patient information. Stable angina patients with inadequate health literacy levels are likely to show higher decisional conflict about their chosen treatment [55]. Concerns were raised about the accessibility of CONNECT to people with low health and digital literacy levels, non-English speakers and ethnic minorities. Only two patient participants from the alpha testing phase had low health literacy levels and volunteers in all study phases were of White British ethnicity. Consultation with underserved communities is needed to inform alternate formats and delivery mechanisms of CONNECT. To improve the development of future PtDAs for any condition and treatment, or screening decisions, patient involvement and user-testing should include a diverse range of people from different social, economic and cultural backgrounds.

## Conclusions

We successfully developed a PtDA (CONNECT) for one of the commonest cardiac interventions, which when alpha tested was generally acceptable, comprehensible, desirable, and usable by patients and health professionals. Potential benefits were improved SDM, informed consent, and patient safety. Evaluation of CONNECT and the potential benefits associated with its use requires evaluation in a clinical trial. Further research is required to understand how CONNECT and other PtDAs can be made accessible to underserved communities. Global awareness of the importance of SDM is increasing but embedding SDM and PtDAs into clinical practice is at an early stage. We recommend that international organisations standardise the provision of PCI probabilistic risk information where possible. To support the international integration of PtDAs and the transition to a SDM culture within clinical practice, we recommend that health professionals invite patients to use a PtDA and encourage patients to share their health values and treatment preferences during the consultation. Using ‘teach-back’ as an educational strategy may also reduce any misconceptions that patients have about their treatment options.

## Supplementary Information

**Additional file 1:** Supplementary material for the development and user-testing of CONNECT.

## Data Availability

All data generated or analysed during this study are included in this published article and its additional files.

## Abbreviations

BCIS: British Cardiovascular Intervention Society
CAD: Coronary artery disease
CONNECT: COroNary aNgioplasty dECision Tool
ESC: European Society of Cardiology
GMC: General Medical Council
HCP: Healthcare professional
IPDAS: International Patient Decision Aid Standards
NHS: National Health Service
NICE: National Institute for Health and Care Excellence
PCI: Percutaneous coronary intervention
PtDA: Patient decision aid
SDM: Shared decision-making
SUNDAE: Standards for UNiversal reporting of patient Decision Aid Evaluation
