# Supplementary material for: Development and user-testing of a digital patient decision aid to facilitate shared decision-making for people with stable angina
Source: BMC Med Inform Decis Mak. 2022 May 27;22:143. doi: 10.1186/s12911-022-01882-x (PMC9137092; doi:10.1186/s12911-022-01882-x)
Supplement: Supplementary file 1 — Additional file 1: Supplementary material for the development and user-testing of CONNECT. [file 12911_2022_1882_MOESM1_ESM.pdf]

Additional File 1: Supplementary material for the development and user-testing of CONNECT.

Contents

1. Table S1: The Standards for UNiversal reporting of patient Decision Aid Evaluation studies (SUNDAE) checklist 2
2. Table S2: Feedback from the co-design workshops. 5
3. Alpha testing cognitive interview guide 9
4. Alpha testing acceptability questionnaire – patient version 10
5. Alpha testing Acceptability questionnaire – health professional version 13
6. Table S3: Data analysis process using deductive content analysis, following an unconstrained matrix approach. 16
7. Table S4: International Patient Decision Aids Standards checklist (version 4) 17
8. Example screenshots of CONNECT prototype 2 21
9. Table S5: Alpha testing illustrative participant quotes 23

Table S1: The Standards for UNiversal reporting of patient Decision Aid Evaluation studies (SUNDAE) checklist

This study reports only on the development and alpha testing of a patient decision aid. Therefore, checklist items 13-15, 17-18 and 21-24 are not applicable to this study as they relate to beta testing and evaluation of a decision aid.

Section/ Topic	Page No.	Item No.	Checklist Item
**Title and Abstract**			
	1	1	Use the term patient decision aid in the abstract to identify the intervention evaluated and, if possible, in the title.
	1	2	In the abstract, identify the main outcomes used to evaluate the patient decision aid.
**Introduction**	**As part of standard introduction (the problem, gaps, purpose)**		
	2	3	Describe the decision that is the focus of the patient decision aid.
	2	4	Describe the intended user(s) of the patient decision aid.
	2	5	Summarize the need for the patient decision aid under evaluation.
	2	6	Describe the purpose of the evaluation study with respect to the patient decision aid.
**Methods**	**Studies with a comparator should also address Items 7-13 for the comparator if possible**		
	3-5, Figure 1,	7	Briefly describe the development process for the patient decision aid (and any comparator), or cite other documents that describe the development process. At a minimum include:<br>• Participation of stakeholders in its development<br>• The process for gathering, selecting and appraising evidence to inform its content<br>• Any testing that was done
	2,4,6, Figures 2 & 3	8	Identify the patient decision aid evaluated in the study (and any comparator) by including:<br>• Name or information that enables it to be identified<br>• Date and/or version number<br>• How it can be accessed, if available
	3	9	Describe the format(s) of the patient decision aid (and any comparator) (e.g. paper, online, video).
	3, Figures 2 & 3	10	List the options presented in the patient decision aid (and any comparator).
	4-6, Figures 2 & 3, Table S2	11	Indicate the components in the patient decision aid (and any comparator) including:

Section/ Topic	Page No.	Item No.	Checklist Item
			• Explicit description of the decision*<br>• Description of health problem*<br>• Information on options and their benefits, harms, and consequences*<br>• Values clarification (implicit or explicit)*<br>• Numerical probabilities<br>• Tailoring of information or probabilities<br>• Guidance in deliberation<br>• Guidance in communication<br>• Personal stories<br>• Reading level or other strategies to help understanding<br>• Other components<br>*These components are needed to meet the definition of a patient decision aid.
	As above	12	Briefly describe the components from Item 11 that are included in the patient decision aid (and any comparator) or cite other documents that describe the components.
	N/A	13	Describe the delivery of the patient decision aid (and any comparator) including:<br>• How it was delivered (e.g. by whom and/or by what method)<br>• To whom it was delivered<br>• Where it was used<br>• When it was used in the pathway of care<br>• Any training to support delivery<br>• Setting characteristics and system factors influencing its delivery
	N/A	14	Describe any methods used to assess the degree to which the patient decision aid was delivered and used as intended (also known as fidelity).
	N/A	15	Describe any methods used to understand how and why the patient decision aid works (also known as process evaluation) or cite other documents that describe the methods.
	2-3	16	Identify theories, models or frameworks used to guide the design of the evaluation and selection of study measures.
	N/A	17	For all study measures used to assess the impact of the patient decision aid on patients, health professionals, organization, and health system:<br>• Identify the measures<br>• Indicate the timing of administration in relation to exposure to the patient decision aid and health care interventions
	N/A	18	For any instruments used:<br>• Name the instrument and the version (if applicable)<br>• Briefly describe the psychometric properties, or cite other documents

Section/ Topic	Page No.	Item No.	Checklist Item
**Results**	**In addition to standard reporting of results**		
	6-7, Table 1	19	Describe the characteristics of the patient, family, and carer population(s) (e.g. health literacy, numeracy, prior experience with treatment options) that may affect patient decision aid outcomes.
	6-7, Table 1	20	Describe any characteristics of the participating health professionals (e.g. relevant training, usual care vs. study professional, role in decision making) that may affect decision aid outcomes.
	N/A	21	Report any results on the use of the patient decision aid:<br>• How much and which components were used<br>• Degree to which it was delivered and used as intended (also known as fidelity)
	N/A	22	Report relevant results of any analyses conducted to understand how and why the patient decision aid works (also known as process evaluation).
	N/A	23	Report any unanticipated positive or negative consequences of the patient decision aid.
**Discussion**	**As part of the standard discussion section (summary of key findings, interpretation, limitations and conclusions):**		
	N/A	24	Discuss whether the patient decision aid worked as intended and interpret the results taking into account the specific context of the study including any process evaluation.
	10-12	25	Discuss any implications of the results for patient decision aid development, research, implementation, and theory, frameworks or models.
**Conflict of Interest**			
	Yes, see competing interests statement (pg. 12)	26	All study authors should disclose if they have an interest (professional, financial or intellectual) in any of the options included in the patient decision aid or a financial interest in the decision aid itself.

Table S2: Feedback from the co-design workshops

Topic	Activity	Summary of feedback	Incorporation of feedback into CONNECT prototype 1
Patient pathway for stable angina	Group discussion with health professionals about where the PtDA (CONNECT) could be used in patient pathway. Use of paper 'flip-board' to draw the patient pathway. Group discussion about potential barriers/ facilitators to using CONNECT.	• Patient pathways differ across UK NHS Trusts; in some hospitals, patients undergo diagnostic coronary angiography first before a treatment decision is made. In other hospitals, patients undergo coronary angiography with the option to proceed immediately to angioplasty during the same procedure, where clinically appropriate.<br>• Provide CONNECT to patients before pre-assessment clinic to complete at home. Patient input to be reviewed by health professional at clinic appointment.	Patients access to CONNECT should be at the point of referral for elective coronary angioplasty and reviewed at home prior to their pre-assessment clinic appointment. Therefore, coronary artery bypass graft (CABG) surgery should be briefly mentioned as a possible future treatment option in CONNECT, but because this is a different decision (which would involve a surgical consultation), the focus of CONNECT would be on medications plus/minus angioplasty. Treatment options to be presented side-by-side to help provide a balanced view.[1]
Format, access, and distribution	Presentation of preliminary ideas for format, access and distribution. Participants voted on their preference.	• Language and accessibility are important factors to consider in the design of CONNECT.<br>• Mixed responses in preferences for different formats.	Readability of CONNECT to be at Grade 8, as recommended by IPDAS. CONNECT prototype format to be an App and website with distribution via a website link.
Multi-media content	Ideas for graphics, videos and logos presented followed by group discussion. Participants voted for their preference.	• Preference for real images, rather than cartoons, and use of stent photograph next to a coin to indicate relative size.<br>• Include links to the British Heart Foundation (BHF) resources.	Photographs of people, a stent and coin, and accurate diagrams were included. External links to BHF and NHS resources were included. The CONNECT logo incorporated simple black lettering and a red heart.

		• Simple CONNECT logo with heart image.	
Risk communication	Patient workshop: Participants divided into two groups. Different formats for communicating risks presented. Participants voted for their preferred format, followed by group discussion facilitated by researcher.	• All risks linked to treatment options should be included and presented as minor or major risks.<br>• There was insufficient data to build an algorithm that accurately calculates individualised procedural risks and prognosis.<br>• There was no consensus on the preferred format for the presentation risks; some preferred icon arrays but others did not.	The risks of coronary angioplasty were divided into common and less common categories. Average population data from British Cardiovascular Intervention society (BCIS) audit was used, as insufficient data was available to develop an algorithm for individualised risk.<br>Probabilistic risk was presented numerically (frequency, 1 in X) and graphically (icon arrays) to account for patient differences in numeracy and graph literacy levels, as recommended.[2] People icons to be used in icon arrays as risk recall is significantly higher than other icon types[2]. Risks were framed in terms of both potential losses (chance of death) and potential gains (chance of survival) and frequencies for each risk presented with the same denominator, as recommended.[3]
Value clarification method (VCM) (i.e. processes used to help patients become aware of how important	Patient workshop: Participants divided into two groups. Example VCM provided for participants to complete. Feedback provided and other factors influencing decision-making discussed in each	• Service users did not understand how to answer the explicit VCM used in the workshop.<br>**Factors influencing decision-making:**<br>• Percentage proportion of the blood vessel that is blocked<br>• Availability of support from partner	There is no consensus on which type of VCM is best to use in PtDAs.[4] Theory-based recommendations for the design of VCMs were used in the development of CONNECT's VCMs.[5] Both implicit VCMs (options presented side-by-side in table format, patient stories) and explicit VCMs (Likert scale to rate importance of topics that are not usually discussed in consultations: support, employment, driving,

particular aspects of each treatment option are to them)	group, facilitated by a researcher.<br>Factors influencing decision-making presented to health professionals followed by group discussion and further suggestions.	• Employment and need to have time off work, especially if self-employed<br>• Uncertainties about complications<br>• Length of hospital stay and recovery period<br>• Likelihood of successful outcome<br>• Impact on holiday plans and ability to drive	hobbies, travel plans, angina symptoms, other medical treatments) were included in CONNECT prototype 1. Patient stories focused on the experience of decision-making and health values, rather than the actual decision made, to avoid bias.
Interactive activities	Ideas for interactive activities presented followed by group discussion and suggestions from patient and health professional participants.	• Use of knowledge quiz to elicit misunderstandings received a mixed response. Some attendees thought patients might feel judged if their knowledge score is low or if they have unhealthy lifestyles, such as smoking. Others liked the idea of having a personalised knowledge score generated from the quiz.<br>• Other suggestions: section for patients to write down questions to ask the doctor/nurse, list of common patient misconceptions about angioplasty treatment.	Instead of a knowledge quiz, the validated 10-item low literacy version of the Decisional Conflict Scale (DCS) was incorporated into CONNECT prototype 1 to encourage patient participation in decision-making.[6] Angina symptom burden is a key consideration in decision-making for coronary angioplasty. Therefore, an angina symptom questionnaire was incorporated into CONNECT. The prototype 1 questionnaire included 4 statements describing angina symptoms of differing severity. Each statement was written in lay language and corresponded with the 4-level Canadian Cardiovascular Society Angina Classification system.[7] A fifth statement indicating no angina symptoms was also added. The patient chooses True, False or Unsure for each of the 5 statements.<br>A ‘free text’ box was included in CONNECT to allow patients to write down questions or

			concerns that they wish to discuss with their doctor or nurse.
Personalised summary	Group discussion about patient and health professional participants' perspectives about the personal summary and where it could be used in clinical practice.	• Some attendees were not interested in this function, whereas others thought it would be useful.	CONNECT generates a personal summary based on patient responses to the explicit value clarifications activity and angina symptoms evaluation. Summary to be used at pre-assessment clinic to help identify areas to focus upon during discussion with patient.

References

1. Abhyankar P, Volk RJ, Blumenthal-Barby J, Bravo P, Buchholz A, Ozanne E, et al. Balancing the presentation of information and options in patient decision aids: an updated review. BMC medical informatics and decision making. 2013;13(2):S6.

2. Zikmund-Fisher BJ, Witteman HO, Dickson M, Fuhrel-Forbis A, Kahn VC, Exe NL, et al. Blocks, Ovals, or People? Icon Type Affects Risk Perceptions and Recall of Pictographs. Medical Decision Making. 2014;34(4):443-53.

3. Trevena LJ, Zikmund-Fisher BJ, Edwards A, Gaissmaier W, Galesic M, Han PKJ, et al. Presenting quantitative information about decision outcomes: a risk communication primer for patient decision aid developers. BMC medical informatics and decision making. 2013;13(2):S7.

4. Witteman HO, Gavaruzzi T, Scherer LD, Pieterse AH, Fuhrel-Forbis A, Chipenda Dansokho S, et al. Effects of Design Features of Explicit Values Clarification Methods:A Systematic Review. Medical Decision Making. 2016;36(6):760-76.

5. Pieterse AH, de Vries M, Kunneman M, Stiggelbout AM, Feldman-Stewart D. Theory-informed design of values clarification methods: a cognitive psychological perspective on patient health-related decision making. Social science & medicine (1982). 2013;77:156-63.

6. O'Connor AM. User Manual - Decisional Conflict Scale (10 item question format). 1993 [updated 2010].

7. Knuuti J, Wijns W, Saraste A, Capodanno D, Barbato E, Funck-Brentano C, et al. 2019 ESC Guidelines for the diagnosis and management of chronic coronary syndromes: The Task Force for the diagnosis and management of chronic coronary syndromes of the European Society of Cardiology (ESC). European heart journal. 2019;41(3):407-77.

Alpha testing cognitive interview guide

Desirability probes

- What did you think of this section of CONNECT [layout, font size, length, easy to use, navigation etc.]?

Usability/ observational probes

- Why did you navigate to [item] first/last?
- Why did you say [word, phrase] when navigating [item]?
- Why did you do [body language] when navigating [item]?
- Why did you change your response in [item]?
- Why did you hesitate in your response in [item]?

Comprehensibility probes

- What do you think the purpose of this section is?
- How understandable did you find the information in this section [probe further on specific items]? (patient participants only)
- How understandable do you think this section will be to your patients [probe further on specific items]? (Health professional participants only)
- What have you read in this section that some patients might find confusing?
- What do you think [item, word, phrase] means in this context? (patient participants only)

Content probes

- Is [item] relevant?
- Is there any aspect missing that will help patients to make a decision about angioplasty?
- Is there any aspect missing that you think patients need to know? (Health professionals only)
- Is there anything that you specifically like about this section?
- Is there anything that you do not like about this section?
- Is there anything that you would like to change about this section?
- Do you have any further comments you would like to add?

Acceptability probes

- Do you think using the tool will help patients feel more involved in the decision-making process (why/ why not)?
- Do you feel the tool will be useful for patients to use during a consultation (why/why not)?
- Do you think the personal summary will be used by patients (why/why not)?
- Do you think this tool would fit into your practice (why/why not)? (Health professional participants only)
- Do you think the tool will help health professionals better understand their patient's values and concerns about their treatment decision (why/why not)?
- Do you have any further comments you would like to add?
- What can we do to improve CONNECT?
- Would you recommend using CONNECT to a colleague/ friend?

Alpha testing acceptability questionnaire – patient version

Introduction: In this short questionnaire we will ask you about your thoughts on using CONNECT. Please **circle one** number for each question

1. **How easy was it to use CONNECT?**

Very difficult<br>1	Fairly difficult<br>2	Neither easy or difficult<br>3	Fairly easy<br>4	Very easy<br>5

2. **How understandable was the information?**

Very difficult<br>1	Fairly difficult<br>2	Neither easy or difficult<br>3	Fairly easy<br>4	Very easy<br>5

3. **How much did you enjoy using CONNECT?**

Very boring<br>1	Fairly boring<br>2	Neither enjoyable or boring<br>3	Fairly enjoyable<br>4	Very enjoyable<br>5

4. **How helpful did you find CONNECT?**

Very unhelpful<br>1	Fairly unhelpful<br>2	Neither helpful or unhelpful<br>3	Fairly helpful<br>4	Very helpful<br>5

5. **Was the amount of time it took to complete CONNECT acceptable?**

Very unacceptable<br>1	Fairly unacceptable<br>2	Neither acceptable or unacceptable<br>3	Fairly acceptable<br>4	Very acceptable<br>5

6. **Overall, how would you rate your satisfaction with CONNECT?**

Very dissatisfied<br>1	Fairly dissatisfied<br>2	Neither satisfied or dissatisfied<br>3	Fairly satisfied<br>4	Very satisfied<br>5

7. **Please rate how useful you found the Facts section.**

No use<br>1	Some use<br>2	Neither useful or no use<br>3	Fairly useful<br>4	Very useful<br>5

8. **Please rate how useful you found the Treatment options section.**

No use<br>1	Some use<br>2	Neither useful or no use<br>3	Fairly useful<br>4	Very useful<br>5

9. **Please rate how useful you found the Things to consider section.**

No use<br>1	Some use<br>2	Neither useful or no use<br>3	Fairly useful<br>4	Very useful<br>5

10. **Please rate how useful you found the About me section.**

No use<br>1	Some use<br>2	Neither useful or no use<br>3	Fairly useful<br>4	Very useful<br>5

11. Please rate how useful you found the my decision section.

No use<br>1	Some use<br>2	Neither useful or no use<br>3	Fairly useful<br>4	Very useful<br>5

12. Please rate how useful you found the my summary section.

No use<br>1	Some use<br>2	Neither useful or no use<br>3	Fairly useful<br>4	Very useful<br>5

Alpha testing Acceptability questionnaire – health professional version

Introduction: In this short questionnaire we will ask you about your thoughts on using CONNECT. Please **circle one** number for each question

1. How easy was it to use CONNECT?

Very difficult<br>1	Fairly difficult<br>2	Neither easy or difficult<br>3	Fairly easy<br>4	Very easy<br>5

2. How understandable was the information/ do you think patients will find the information?

Very difficult<br>1	Fairly difficult<br>2	Neither easy or difficult<br>3	Fairly easy<br>4	Very easy<br>5

3. How much did you enjoy using CONNECT?

Very boring<br>1	Fairly boring<br>2	Neither enjoyable or boring<br>3	Fairly enjoyable<br>4	Very enjoyable<br>5

4. How helpful did you find CONNECT/ do you think patients will find CONNECT?

Very unhelpful<br>1	Fairly unhelpful<br>2	Neither helpful or unhelpful<br>3	Fairly helpful<br>4	Very helpful<br>5

5. Was the amount of time it took to complete CONNECT acceptable?

Very unacceptable 1	Fairly unacceptable 2	Neither acceptable or unacceptable 3	Fairly acceptable 4	Very acceptable 5

6. Overall, how would you rate your satisfaction with CONNECT?

Very dissatisfied 1	Fairly dissatisfied 2	Neither satisfied or dissatisfied 3	Fairly satisfied 4	Very satisfied 5

7. Please rate how useful you found the Facts section.

No use 1	Some use 2	Neither useful or no use 3	Fairly useful 4	Very useful 5

8. Please rate how useful you found the Treatment options section.

No use 1	Some use 2	Neither useful or no use 3	Fairly useful 4	Very useful 5

9. Please rate how useful you found the Things to consider section.

No use 1	Some use 2	Neither useful or no use 3	Fairly useful 4	Very useful 5

10. Please rate how useful you found the About me section.

No use 1	Some use 2	Neither useful or no use 3	Fairly useful 4	Very useful 5

11. Please rate how useful you found the my decision section.

No use<br>1	Some use<br>2	Neither useful or no use<br>3	Fairly useful<br>4	Very useful<br>5

12. Please rate how useful you found the my summary section.

No use<br>1	Some use<br>2	Neither useful or no use<br>3	Fairly useful<br>4	Very useful<br>5

Table S3: Data analysis process using deductive content analysis, following an unconstrained matrix approach

Analysis steps	Process
1. Familiarisation	All interview transcripts and field notes of interview observations were read and ideas for codes and categories noted by the lead analyst (EH).
2. Development of categorisation matrix	A categorisation matrix consists of themes, categories, and codes. The main interview questions formed the matrix themes:<br>1. Acceptability: Ways in which CONNECT could potentially add value to patients, health professionals and health services<br>2. Usability: The degree to which CONNECT was accessed, navigated, and completed<br>3. Comprehensibility: The degree to which the content of CONNECT was understandable<br>4. Content: Perspectives on the usefulness and factual accuracy of CONNECT's content<br>5. Desirability: The degree to which CONNECT was presented in a visually appealing way<br>Ideas for codes and categories, noted in step 1, were added to the categorisation matrix.
3. Testing the categorisation matrix	The initial matrix was applied to three transcripts and several codes and categories were revised. Changes were made based on the principles of an unconstrained matrix; the codes can change and move between categories, within the pre-determined themes. Three additional transcripts were read by a second researcher (FA) and key categories noted. The coders discussed their findings to develop the final categorisation matrix.
4. Applying the final categorisation matrix	The final categorisation matrix was applied to all interview transcripts and observation field notes.
5. Summarising data	Data within the categorisation matrix were summarised with illustrative quotes and presented as a narrative.

Table S4: International Patient Decision Aids Standards checklist (version 4)

Category	Code	Item	Location
**Qualifying**	Q1	Describes health condition or problem for which index decision is required	Stable angina symptoms caused by coronary heart disease in section “My heart and symptoms”.
	Q2	Explicitly states decision under consideration (index decision)	Coronary angioplasty and medicines or medicines only described in sections “My heart and symptoms”, “My options” and “My decision”.
	Q3	Describes the options available for the index decision	Full explanation given in text and video in section “My options”.
	Q4	Describes the positive features of each option	Explains potential benefits in side-by-side comparison table in section “My options”.
	Q5	Describes the negative features of each option	Explains potential risks and side effects in section “My options”.
	Q6	Describes the features of options to help patients imagine the physical, social and/or psychological effects	Describes answers to frequently asked questions to help patients imagine the physical, social and/or psychological effects for both options, in section “Personal stories”. “My options” section also presents physical effects of both options.
**Certification**	C1	Shows positive and negative features of options with equal detail	Yes, in “My options” section.

	C2	Provides information about the funding source used for development	Yes, in “About” section.
	C3	Provides citations to the evidence selected	Yes, in “About” and “My options” sections.
	C4	Provides a production or publication date	Yes, in “About” section.
	C5	Provides information about update policy	Yes, in “About” section.
	C6	Provides information about the level of uncertainty around outcome probabilities	Yes, in “My options” section.
**Quality**	QA1	Development included needs assessment to determine what patients need to make the decision	Yes, previous research and co-design workshops identified decisional needs.
	QA2	Development included needs assessment to determine what health professionals need to discuss decision	Yes, previous research and co-design workshops identified decisional needs.
	QA3	Development included review by patients not involve in producing the DSI	Yes, different people involved at different stages. A total of 34 patients/service users included. See Figure 1 flow-diagram in manuscript.
	QA4	Development included review by professionals not involve in producing the DSI	Yes, different people involved at different stages. A total of 29 health professionals included. See Figure 1 flow-diagram in manuscript.

	QA5	DSI was field tested with patients facing the decision	N/A to be evaluated in future study.
	QA6	DSI was field tested with practitioners who counsel patients facing the decision	N/A to be evaluated in future study.
	QA7	Includes author/developers' credentials or qualifications	Yes, in "About" section.
	QA8	Evidence that DSI improves match between patient preferences and chosen option	N/A to be evaluated in future study.
	QA9	Evidence that DSI helps patient improve knowledge about options' features	N/A to be evaluated in future study.
	QA10	Describes how research evidence was selected/synthesized	Yes, described in methods section of manuscript.
	QA11	Describes the quality of research evidence used	Clinical evidence reviewed by Steering Group members.
	QA12	Provides step by step way to make decision	Yes, sections numbered in home page of CONNECT.
	QA13	Includes tools to use when discussing options with practitioner	Yes, CONNECT generates a personal summary of patient's responses that can be used during a consultation with a health professional.

	QA14	Describes the natural course of the condition	Not explicitly provided.
	QA15	Makes it possible to compare features of available options	Yes, side-by-side comparison table of the two treatment options provided in “My options” and “Personal stories” sections.
	QA16	Reports readability levels	Not stated in CONNECT. Data available upon request from authors.
	QA17	Provides information about outcome probabilities (OPs)	Yes, in “My options” section.
	QA18	Specifies reference class of patient for which OPs apply	Yes, in “My options” section.
	QA19	Specifies event rates for Ops	Yes, in “My options” section.
	QA20	Specifies the time period over which OPs apply	Yes, in “My options” section.
	QA21	Allows to compare OPs using the same denominator	Yes, in “My options” section.
	QA22	Provides more than one way of viewing probabilities	Yes, text and icon arrays in “My options” section.
	QA23	Asks patients to consider which positive and negative features matter most to them	Yes, video and ‘field’ for patients to report their values in “What matters to me” section.

Example screenshots of CONNECT prototype 2
PCI procedural risks and Personal Stories

CONNECT   BACK   HOME

How might treatment with coronary angioplasty and stents help me?

What are the risks of the procedure?

Most medical treatments carry some risk of complications. The risks of a coronary angiogram and coronary angioplasty vary from patient to patient. Older people and those with more severe heart disease or other medical conditions (e.g. kidney disease and diabetes) are at a higher risk. Your doctor or nurse will talk to you in more detail about your own risk. The possible complications of the procedure are listed below. The numbers shown for each risk were calculated from people who have had this procedure. **Remember that your individual risk of a complication could be higher or lower than the numbers shown.** Click on each complication listed below to find out more about the risk.

Damage to the blood vessel or bleeding that won't stop at the wrist or groin

Heart attack

There is a risk of having a heart attack during or shortly after the procedure.

For every **1000** people who had a coronary angioplasty*

990 people did NOT have a heart attack   **10 people** had a heart attack

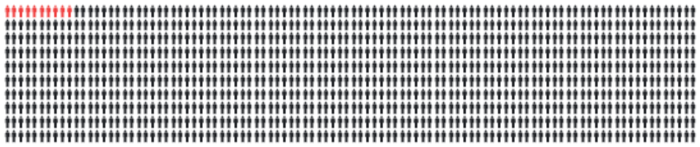

10 people had a heart attack
990 people did NOT have a heart attack

Click here to hide the picture

** Based on data (MI related to PCI; primary definition) from the ISCHEMIA research study (Chaitman et al., 2020; doi.org/10.1161/CIRCULATIONAHA.120.047987)*

Damage to the kidneys

Death

Allergic reaction to the dye

Emergency open heart surgery

Stroke

BACK   HOME   NEXT OPTION

CONNECT

BACK HOME

Personal stories

Relationships and support

Photo of 'patient'

"I have a young family and my husband works away a lot. It's been difficult to keep up with two young boys. I get tired quite easily. I was worried about how I would cope if my angina got worse. On the other hand, if I chose to have the coronary angioplasty, I had to think about what arrangements I would need to put into place in terms of support and child care."

0:00 / 0:26

What support will I need?

Coronary angioplasty and stents plus 'heart protective' medicines

You will need to arrange transport home after the coronary angioplasty treatment, as you will not be able to drive. If possible, arrange for someone to stay with you for the first 24 hours when you go home.
After having coronary angioplasty and stents, your healthcare team may refer you to cardiac rehabilitation, if it is available. Cardiac rehabilitation is an important part of your treatment and recovery and you should try to attend all classes.

'Heart protective' medicines only

It is common to feel tired, worried or a bit down about having heart problems. It is important to have support from your family, friends or carers. The British Heart Foundation and your GP and heart nurse are important sources of support.

When is it safe to have sex?

Sex is just another form of exercise and no more likely to trigger a heart problem than any other physical activity. If you start to get angina symptoms during sex, you should stop, rest and take your GTN spray or tablets if needed.

Coronary angioplasty and stents plus 'heart protective' medicines

You should wait until your puncture wound has healed before having sex. This might take a few days (wrist wound) or at least one week (groin wound).

'Heart protective' medicines only

You can continue to have sex when you feel well enough to do so.

BACK NEXT PERSONAL STORY

Table S5: Alpha testing illustrative participant quotes

Categorisation matrix with illustrative quotes from alpha testing of CONNECT prototype 1

Themes	Categories & Codes	Illustrative quotes
1. Acceptability	**Category 1.a. Facilitating shared decision-making:**<br>• Empowering and informing patients	**Nurse 11:** "I mean it gives them a lot of information. I know the doctors don't have a lot of time to explain risks and benefits and that sort of thing in their clinic letters because they've got to take a history, they've got to take a medication history. So perhaps this app makes people a little bit more informed and aware that there are options for them."
	• Indicating preferred treatment	**Patient and partner 7:**<br>**Partner:** "Which treatment option do you prefer? Coronary angiography, or angioplasty, medication, or lifestyle change, no treatment, or unsure."<br>**Patient:** "That again depends on what you've been told."<br>**Partner:** "Exactly, because it's the medic that"<br>**Patient:** "It's them that rule it."<br>**Partner:** "Well that exactly."<br>**Patient:** "It's alright saying oh my heart's goofed so I want an angioplasty and the doctor turns round oh no you're not, you've having tablets. That's up to the…"<br>**Partner:** "Yeah it's in the lap of the gods isn't it really."
	• Understanding patient preferences, values and concerns	**Nurse 3:** "I think so because it included a lot of things that we might think of as secondary almost, like the hobbies or the fact that they're worried about flying on holiday. You know, you can't fly for a week, you can be quite blasé about it, but people's priorities might not be what your priorities are. So, you try and swap and change and see it from their perspective a little bit more."

Themes	Categories & Codes	Illustrative quotes
	• Ease of access to consistent and credible information	**Patient 6**: "Just thinking back it would have given me, yeah, a resource or something to, more structured resource, rather than what I've done, which is just randomly hunting the internet, googling stuff and coming up with websites and such like, which yeah, sometimes you just don't know what you can trust."
	• Patient recall and reassurance	**Patient 14:** "Well I'd find it quite reassuring to have a record, either printed or on the computer of what I had said because if I was thinking afterwards what did I say to the question, I don't remember now. So, I could look back."
	• Time for deliberation	**Patient 6:** "If the sort of consultant has given you the options, you can go away and look up, use this to help with further information and digest it all"
	**Category 1.b. Improving care processes:** • Ensuring consent is informed and voluntary	**Nurse 13**: "Again I think this bit is probably most useful for people undertaking the pre-assessment and giving them some sort of idea about what they're thinking and what advice I might need to give them extra to help them to make an informed decision."
	• Personalising pre-assessment clinic consultations	**Nurse 11:** "As I say I find it, the, our intentions hopefully with telephone pre-assessments is to cut down on the time of the pre-assessment, so we can maybe fit more in. So, if you have more information that you can then target, as I say, towards specific concerns or anything like that, its leading you there already and without having to get a convoluted explanation from a patient. You can go ah I can see you're a little bit concerned about something. So yeah, I think I probably would use it, it would be quite helpful to help me understand how people are feeling coming up to treatment. So, whether they're deciding more one way or another towards one method of treatment."

Themes	Categories & Codes	Illustrative quotes
	**Category 1.c. Quality and safety in practice:**<br>• Patient safety	**Nurse 12:** "I was just thinking the only, if I saw this, this screen and I saw that this patient is getting angina symptoms practically all the time, I might be concerned and I might try to expedite their procedure. So, it might help in that way."
	• Audit of consent process and practices	**Cardiologist 4:** "Well it lends itself to, I mean I don't know if this information is, would be accessible to the cardiologist or the hospital to use for audit purposes, because if it is and if lots of patients are saying I'm unsure about something, then it will allow us to change the patient pathway potentially. But there are some sort of quality improvement angles."
**2. Usability**	**Category 2.a. Accessibility:**<br>• Login process*<br>• Digital literacy	**Cardiologist 1:** "Yeah the only thing I would have said on there and I had to stop and think of, was I had to put the slash in as well. Whereas on a normal screen you would have the date of birth, you put the first two digits in and it would automatically switch to the secondary digits automatically, if you see what I mean."<br>**Patient 9:** "Its fine, but I think not everybody is au-fait with modern technology and presumably heart complaints are more for the older person and so you might get some old lady or gentleman who aren't au-fait and might not have, might even not have a computer, I don't know. I mean I know most people have, but not everybody does do they?"
	**Category 2.b. Navigation:**<br>• Handling of iPad*<br>• Navigating between sections*<br>• Navigating between CONNECT and external websites*<br>• Navigating within each section*<br>• Identifying the link to risk icon arrays	**Patient 5:** "The only thing that I got a little bit lost and I had to look around for was the back button, maybe the back button should go at the bottom".<br>**Patient 6:** "It's quite good. My next question would then be how on earth do you get back to where you were once you've viewed."<br>**Researcher:** "Have you seen that you can click on each of them [risk icon arrays]?"<br>**Nurse 11:** "No, I hadn't, but, oh right okay, oh that's quite useful. Yeah, I thought that that was just like another bullet point. So maybe you can make the information icon slightly bigger and an explanation that that could lead you to, or do more information or something next to each of those.

Themes	Categories & Codes	Illustrative quotes
	**Category 2.c. Functionality:**<br>• E-mail summary function<br>• Missing functions and capabilities<br>• Time to complete<br>• Technical problems*<br>• Completion of interactive elements*	**Cardiologist 1:** "But then you've got to choose, about transferring confidential information."<br>**Cardiologist 15:** "Well I was just wondering, I mean it's probably too fancy, but it you had, if it was an algorithm. So, if you answer this, if you answer these as agree or strongly agree, then the discussion is just about those things, they're not about the other things that you maybe weren't that fussed about. So, then you're tailoring that discussion about the certain, the individual aspects that that person is worried about."<br>**Patient 5:** "So unless you want to put additional links in there, my only concern would be is if people are pushed for time, it might be, right at the beginning, you might just want to say this might take approximately an hour, because there's always a danger somebody picks this up and thinks oh it's only going to take me five minutes, but actually when you get into it there's a lot of detail here."
**3. Comprehensibility**	**Category 3.a. Language:**<br>• Health literacy and use of plain language	**Patient 14:** "I thought it was understandable. I don't, I don't think you'd really want more than that because it would start to look too wordy."
	• Languages other than English	**Nurse 13:** "Yeah, I mean for our population at (Name of Place), we have a lot of Asian, you know, we have a high Asian population. So different languages would be helpful, definitely."
	**Category 3.b. Interpretation of information:**<br>• Decision score	**Researcher:** "What do you think this 'my scoring' section is telling you?"<br>**Patient 7:** "All gobbledygook."
	• Clarity	**Researcher**: "Is it clear what you need to do?"<br>**Patient 6**: "Well I'm going to hope for, I would hope it would open. The answer to that is no it's not clear."

Themes	Categories & Codes	Illustrative quotes
	• Risk communication	**Patient 5:** "Well again I'm a great believer in pictures, percentages, yeah, fifty percent, but you can't visualise it. Where, obviously when you have it in front of you like that, then you can see right up there, yes, one in two, is pain, yeah, yeah."
**4. Content**	**Category 4.a. Accuracy of content:** • Heart disease facts	**Nurse 11:** "But it all sounds like it's quite useful. It's got that good explanation of what the disease is and the disease process. It gives people a bit of clarification as to this, there might be some symptoms that I'm getting, perhaps it's the right thing to do, to come in and get this investigated."
	• Medicines	**Cardiologist 4:** "Well I certainly wouldn't include stroke, that's completely irrelevant. There's absolutely no evidence that Nicorandil will reduce the risk of stroke. If I'm being critical, that's factually wrong."
	• Lifestyle changes	**Patient 9**: "We all know about alcohol and weight puts a strain on your heart and I know about that. But its, it is good to see it in black and white. It just makes, it focuses the mind, you know."
	• Coronary angioplasty risks	**Cardiologist 4:** "Oh my god, that's a very controversial statement when you put it straight out there and you've said it's likely to have no effect on whether you have a heart attack in the future. Okay. I think the problem you're gonna have by asking that question and making a very categorical statement like what you've said, it is likely to have no effect on whether you have a heart attack in the future, is that again some doctors may not like it and may refuse to use your decision aid because I don't think that that is entirely accurate."
	**Category 4.b. Balanced view of treatment options:** • Treatment comparison	**Nurse 10:** "I really like that you've got option a and option b and then your comparison, that's really good and its nice with the pictures and an explanation at the top of what can, just what you're supposed to do."

Themes	Categories & Codes	Illustrative quotes
	• Coronary artery bypass graft as a treatment option	**Patient 5:** “Because in my mind I went for an angiogram and I was presented with do nothing, sorry medication, go away and think about it, which I suppose is not really an option, stenting, or bypass. So, to me that was maybe a critical one to include”.
	• No treatment as an option	**Cardiologist 8:** “The medical treatment is not an option. Medical treatment is compulsory. So, I would not even say medical treatment is an option. The option is revascularisation. Medical treatment is compulsory for everybody.”
	**Category 4.c. Personalisation of decision aid:**<br>• Decisional Conflict Scale	**Patient 9:** “It’s not an exam I’m doing, I don’t want to be tested on whether I’ve read it all properly.”
	• Angina symptom questionnaire	**Patient 16:** “I think, I think that I’m erm contradicting myself because what it’s, what it’s saying is I get angina symptoms at rest. No, I don’t, never have done. I get angina symptoms when I walk along the street or climb one flight of stairs, yes, I do. So, I do get angina, but not as bad as I did.”
	• Personalised summary	**Consultant 4:** “The summary should be a true summary. It’s just not, because at the moment the summary is basically about me plus my choice added together. I don’t think that’s good enough.”
	**Category 4.d. Value elicitation method:**<br>• Relevance of patient stories	**Patient 6:** “For me personally it’s been, I think it relates to the age profile, is I’m relatively young compared with most other heart patients and I have a young family and it’s been my ability to keep up with two young boys has been impacted by the angina. So, say something related to sort of family, impact on family activities maybe, would be for me personally.”

Themes	Categories & Codes	Illustrative quotes
	• Values statements and concerns box	**Patient 5:** "I'm answering this as if I was going in for, so I'm sort of thinking of the second time I went in, prior to that. I'm just a little bit ambiguous about this question here, is, because I haven't got any travel plans, so actually, what's the options, so I disagree with that, but I am concerned about future travel plans and the cost of insurance. So actually, I agree with that."
**5. Desirability**	**Category 5.a. Presentation:** • Font	**Cardiologist 4:** "You could have a couple of paragraphs in it, as I've said throughout, if you have the option of increasing the font size for some patients, you hit a button and it says increase font size, that would be better for older patients".
	• Colour	**Cardiologist 1:** "I like the colours, I like white background, I like the red, the contrasts are good."
	• Icons and buttons	**Patient 6:** "So keeping it simple and straightforward and clearly different symbols as well. There's no ambiguity between those symbols, six symbols, which helps, once you do get used to them, as to what they are."
	• Layout	**Patient 14:** "Yeah well again its very straightforward for someone who's used to using dropdown boxes."
	**Category 5.b. Use of multimedia:** • Existing multimedia	**Nurse 11:** "Nurse (11): "Like that they've got a picture as well, sort of the visual cues for some people make it a lot more understandable."
	• Required multimedia	**Consultant 15**: "As many pictures and videos as possible really because that's what interests you isn't it, well it would me. So, you could even say less text but more kind of, more pictures, more visual"

*Field notes from interview observations have informed this code
